# Ceramide and Regulation of Vascular Tone

**DOI:** 10.3390/ijms20020411

**Published:** 2019-01-18

**Authors:** Angel Cogolludo, Eduardo Villamor, Francisco Perez-Vizcaino, Laura Moreno

**Affiliations:** 1 Department of Pharmacology and Toxicology, School of Medicine, University Complutense of Madrid, Instituto de Investigación Sanitaria Gregorio Marañón (IiSGM), Ciudad Universitaria S/N, 28040 Madrid, Spain; fperez@med.ucm.es (F.P.-V.); lmorenog@med.ucm.es (L.M.); 2 Ciber Enfermedades Respiratorias (CIBERES), 28029 Madrid, Spain; 3 Department of Pediatrics, Maastricht University Medical Center (MUMC+), School for Oncology and Developmental Biology (GROW), 6202 AZ Maastricht, The Netherlands; e.villamor@mumc.nl

**Keywords:** ceramide, vascular, sphingolipids, reactive oxygen species, oxygen sensing, pulmonary, hypoxic pulmonary vasoconstriction, normoxic ductus arteriosus

## Abstract

In addition to playing a role as a structural component of cellular membranes, ceramide is now clearly recognized as a bioactive lipid implicated in a variety of physiological functions. This review aims to provide updated information on the role of ceramide in the regulation of vascular tone. Ceramide may induce vasodilator or vasoconstrictor effects by interacting with several signaling pathways in endothelial and smooth muscle cells. There is a clear, albeit complex, interaction between ceramide and redox signaling. In fact, reactive oxygen species (ROS) activate different ceramide generating pathways and, conversely, ceramide is known to increase ROS production. In recent years, ceramide has emerged as a novel key player in oxygen sensing in vascular cells and mediating vascular responses of crucial physiological relevance such as hypoxic pulmonary vasoconstriction (HPV) or normoxic ductus arteriosus constriction. Likewise, a growing body of evidence over the last years suggests that exaggerated production of vascular ceramide may have detrimental effects in a number of pathological processes including cardiovascular and lung diseases.

## 1. Introduction

### 1.1. Synthesis and Metabolism

**Figure 1:**
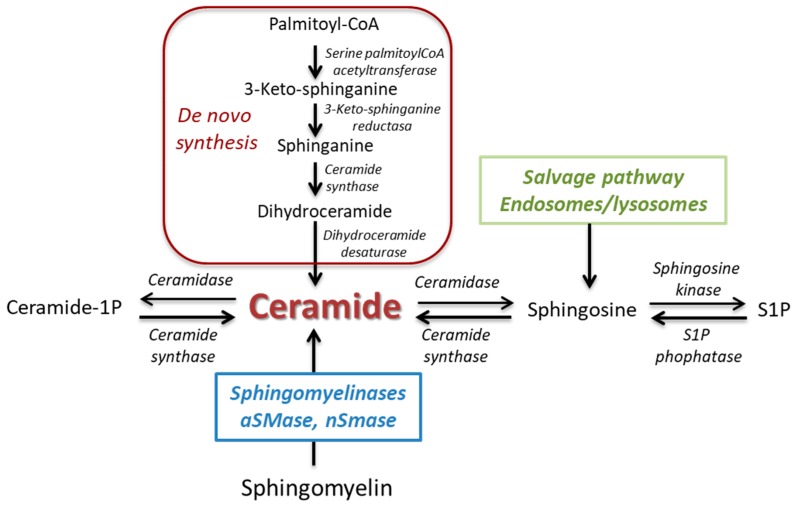
Synthesis and metabolism of ceramide.

Sphingolipids are essential constituents of organelle and cell membranes that also play a key role in signal transductions of crucial physiological processes such as growth, differentiation, proliferation, migration, apoptosis, and cell death [1,2]. Ceramide represents a key point as the core of sphingolipid metabolism with a well-recognized involvement in many cellular functions. Ceramide can be generated through (Figure 1): (1) de novo pathway that synthesizes ceramide from serine and palmitoyl-CoA; (2) the activity of ceramide synthase; or (3) hydrolysis of sphingomyelin, catalyzed by a family of enzymes known as sphingomyelinases (SMases), which are classified into neutral SMases (nSMase 1, 2, and 3), acid SMase (aSMase), and alkaline SMase [1,3,4]. Once generated, ceramide can be hydrolyzed through the acid ceramidase giving rise to sphingosine, which in turn can be phosphorylated to sphingosine 1 phosphate (S1P) due to the action of sphingosine kinases (SK1 and SK2).

### 1.2. The Ceramide/S1P Rheostat

Among the different sphingolipids, most research has focused on the bioactive S1P and ceramide. S1P acts as an autocrine or paracrine mediator by binding to specific membrane receptors (S1P_1–5_), of which S1P_1–3_ are expressed in the cardiovascular system [5,6]. Thus, S1P also exerts a wide range of cellular actions at the vascular level that depend on the type of receptor activated. These include proliferation, migration, and angiogenesis [7]. Likewise, S1P produces endothelium-dependent vasodilatation (via nitric oxide—NO) at low concentrations (nanomolar range), although at higher concentrations (1–100 µM), such as those reached in the presence of thrombi, it produces vasoconstriction [8]. Ceramide has a prominent role in the regulation of programmed cell death (apoptosis) induced by tumor necrosis factor alpha (TNF-α) or FAS ligand [9]. In addition to apoptosis, ceramide has been implicated in endothelial oxidative stress, growth inhibition, cytoskeleton changes, senescence, and vascular tone regulation [1,2,10,11,12,13,14,15]. Thus, ceramide and S1P are bioactive sphingolipids with opposite cell functions, which has led to the proposal of a ‘sphingolipid rheostat’, according to which the cell fate (death/survival) is determined by the balance between ceramide and S1P [2]. Both ceramide and S1P are thought to contribute to vascular tone regulation under physiological and pathological conditions [16]. While the role of S1P in vascular tone regulation has been the subject of a number of review articles [5,17,18,19], attention on ceramide has been disregarded. Thus, this review aims to delve deeper into the regulation of vascular tone by ceramide, with special emphasis on signal transduction pathways in endothelial and smooth muscle cells, leading to the activation of vasorelaxation and vasoconstriction responses, interaction with reactive oxygen species (ROS), involvement in oxygen sensing, and roles in pathological processes.

## 2. Ceramide as Signaling Mediator Regulating Vasomotor Function

The vasoactivity of ceramide has been investigated in several vascular preparations (Table 1). Common strategies in these studies to test the effects of increased cellular ceramide levels include the addition of exogenous cell-permeable ceramide analogs and the application of bacterial SMase from *Bacillus cereus* that cleaves membrane sphingomyelin and releases endogenous ceramide. Vasodilator, vasoconstrictor, or no response have been reported using either of these approaches (Table 1).

### 2.1. Ceramide-Induced Vasodilation

Several studies have shown a vasodilator effect induced by ceramide, however the mechanisms involved and the potential endothelial dependency remains controversial. A few studies have reported that vasodilation to ceramide is, at least partly, endothelium-dependent [21,34] and could involve the activation of endothelial nitric oxide synthase (eNOS) and subsequent NO generation [35]; even when most evidences point toward an impairment of endothelium-dependent vasodilation by ceramide (see Section 4.1). Nevertheless, vasodilation induced by ceramide and sphingomyelinase has also been found in endothelium denuded rat thoracic aortic rings [23,36] and rat mesenteric microvessels [20]. Inhibition of the RhoA/Rho-kinase pathway or activation of guanylyl cyclase and charybdotoxin-sensitive K^+^ channels were proposed to contribute to these relaxant responses.

### 2.2. Ceramide-Induced Vasoconstriction

There is a number of studies indicating the ability of ceramide to promote vasoconstriction. Zheng T et al. [25] reported that nSMase, C8 ceramide and C16 ceramide induced a sustained vasoconstriction of canine cerebral arterial rings in a concentration-related manner that was endothelium independent. These responses were inhibited under Ca^2+^ free conditions and by verapamil and nimodipine, suggesting a role of Ca^2+^ entry through L-type Ca^2+^ channels in ceramide-induced contraction. Interestingly, other ceramides tested in this preparation such as C6, C24:1, and C24:0 lacked vasoconstrictor effect [25] and C2-ceramide even attenuated vasoconstriction [37]. Inhibition of K^+^ channels, which in turn depolarizes membrane potential leading to opening L-type Ca^2+^ channels, has been linked to ceramide-induced vasoconstriction in several studies. Thus, C2-ceramide was shown to inhibit calcium-activated K^+^ channels (KCa) channels and reduced the diameter of isolated perfused small bovine coronary arteries [24]. In rat, chicken, and human resistance pulmonary arteries, SMase and C6-ceramide induces contraction though PKCζ-dependent inhibition of Kv channels (Kv1.5 and Kv2.1) [28,29], which involves the formation of a signaling complex PKCζ-p62-Kvβ [28,38,39] and is also associated with elevation of ROS [26]. These responses were also inhibited by nifedipine, which is consistent with a subsequent activation of L-type Ca^2+^ channels following Kv channel inhibition and depolarization. Additional mechanisms such as increase in calcium sensitization via Rho kinase activation and enhanced calcium entry through transient receptor potential canonical 6 (TRPC6) [27,40] have also being involved in ceramide-induced pulmonary vasoconstriction. Importantly, nSMase-derived ceramide has been reported to potentiate vascular constriction [30] and to contribute to the vasoconstrictor responses induced by thromboxane A_2_ (TXA_2_) [28], angiotensin II [41], and by changes in O_2_ tension (see Section 5) [27,29,40].

In summary, the vascular effects of ceramide appear complex and somewhat puzzling. A number of factors may account for this variability including: (1) Differences in the strategy to enhance ceramide content (exogenously added vs. endogenous); (2) the interaction with different cell types (endothelial vs. smooth muscle cells); (3) the vascular diameter (conductance vs. resistance arteries); (4) the vascular territory (pulmonary vs. cerebral vs. coronary vs. mesenteric arteries); (5) the animal model; (6) the possible conversion of ceramide to other active sphingolipids such as S1P, which can lead to additional complexity; and (7) remarkably, the ceramide analogue tested (short-C2 vs. long C16). Many studies use short-chain ceramides (C2-ceramide and C6-ceramide) mainly because they are water-soluble and permeable to the cell membrane, unlike the natural long-chain ceramides. C2-ceramide is not suitable for mechanistic studies because it does not exist in plasma and shows different properties in comparing to C16-ceramide. The use of exogenous C6-ceramide may be a more adequate tool because, unlike C2-ceramide, it may induce the generation of endogenous long-chain ceramide (C16-ceramide), which was attributed to the recycling of the sphingosine backbone of C6-ceramide via deacylation/reacylation [3]. Nevertheless, the results obtained with long chain ceramides are expected to be more representative since they are more abundant, and their levels are altered in a number of pathological conditions (Table 2, Table 3 and Table 4).

## 3. Ceramide and Redox Signaling

ROS, including both free radicals (such as superoxide-O_2_^−^) and non-radical species (such as hydrogen peroxide-H_2_O_2_), play an essential role in the regulation of physiological and pathophysiological processes within the cardiovascular system [42,43,44,45]. In vessels, NADPH oxidases (Nox), mitochondrial electron transport chain (ETC), xanthine oxidase, and uncoupled endothelial NO synthase (eNOS) are considered main sources of ROS [43,45]. In addition to their role in proliferation, autophagy, or cytotoxicity, ROS are important modulators of vascular tone [43,44,45,46]. The activation of a variety of redox-sensitive signaling pathways comprising protein kinases, phosphatases, channels, and transporters may contribute to ROS-induced modulation of vascular smooth muscle tone. Remarkably, ROS can function as second messengers to mediate, or interfere with, the vasomotor effects of main vasoactive factors such as NO, prostacyclin, angiotensin II, endothelin-1, and TXA_2_ [44,45,47]. In addition, it is well recognized that oxidative stress due to exaggerated ROS production or reduced antioxidant capacity may contribute to vascular diseases [44,45,46]. During the last decade accumulating evidence links ROS signaling either upstream or downstream ceramide production in vascular tissues [11,48,49,50,51].

### 3.1. ROS-Induced Ceramide Production

The ability of ROS to activate different ceramide generating pathways has been extensively studied in many cell types [52,53,54,55]. Thus, a number of reports have indicated that the generation of ROS in response to various stimuli such as antileukemic agents [56], stress [57], ischemia-reperfusion [58], pathogens [59], or hypoxia [26,27] may lead to ceramide accumulation. Both nSMase and aSMase are redox-sensitive enzymes whose activities are incremented by ROS [51,53,55]. Accumulation of ROS triggers the activation of aSMase in neutrophils [54], platelets [60], and splenocytes [61]. Likewise, activation of nSMase by ROS has been reported in vascular smooth muscle cells, fibroblasts, and airway epithelial cells [26,62,63]. Interestingly, SK1 an enzyme mediating the metabolic flow from ceramide to S1P is also redox-sensitive. Thus, ROS production may lead to a misbalance in the ceramide/S1P rheostat in either direction. Recently, Cinq-Frais et al. [62] showed that whereas low H_2_O_2_ concentrations trigger activation of nSMase2 and SK1 and subsequently S1P increase, high H_2_O_2_ levels inhibit SK1 leading to an increase in ceramide content. ROS-induced activation of this nSMase2/SK1 pathway has been also involved in the angiogenic signaling induced by oxidized LDL [64].

### 3.2. Ceramide-Induced ROS

Ceramide triggers the generation of ROS and increases oxidative stress in many mammalian cells and animal models [33,50,51,65]. In vascular cells or tissues, ceramide is known to enhance ROS production as a result of the activation of different redox enzymes including mitochondrial respiratory chain, NADPH oxidase, and uncoupled eNOS [51,65,66]. In vitro studies using isolated mitochondria [67] or endothelial cells [68,69] have suggested a role of ceramide in the mitochondrial ROS production induced by TNF-α or circulating microparticles. Recently, it has been shown that long term exposition to ceramide leads to the increased formation of mitochondria-derived H_2_O_2_ in human resistance arterioles [70]. The cross talk between ceramide signaling pathway and NADPH oxidase-derived ROS has been reported in several vascular cells including rat [26] and human [71] vascular smooth muscle cells and endothelial cells [65,72]. Activation of NADPH oxidases has been associated with the formation of ceramide-enriched membrane rafts (MR). Thus, upon stimulation, ceramide-enriched MRs can be clustered to recruit NADPH oxidase subunits and form redox signaling platforms. In endothelial cells aSMase activates MR clustering to form redox signaling platforms in response to Fas ligand or TNF-α [73,74]. Similar redox signaling platforms following nSMase activation has been shown in vascular smooth muscle cells [26].

### 3.3. Feedforward Amplifying Mechanism

As stated above, many studies point to a clear link between ROS and ceramide in which ROS are able to stimulate ceramide-generating enzymes and, conversely, the accumulation of ceramide triggers the production of ROS. These evidences support the view of the existence of feed-forward mechanism between these two key players [49,50]. Jaffrezou et al. [75] reported a positive feedback loop in which ceramide activated nSMase in myeloid leukemia cells. In endothelial cells, aSMase activates lipid raft clustering to form redox signaling platforms, where O_2_^−^ production enhances aSMase activity, and thereby results in a forwarding amplification of redox signaling [73]. In pulmonary artery smooth muscle cells, Frazziano et al. [26] showed that H_2_O_2_ increases nSMase-derived ceramide production and, conversely, that ceramide increases H_2_O_2_, in line with the existence of a positive feedback cycle. Thus, a feedforward mechanism, which may involve aSMase- or nSMase-derived ceramide [50], leads to an amplification of ROS signaling which is thought to modulate vasomotor and endothelial function [26,46,50,73,76].

## 4. Ceramide and the Endothelium

### 4.1. Ceramide and Endothelial Dysfunction

The endothelium plays an essential role in the control of vascular tone via the production of vasoactive factors [77,78]. Endothelial dysfunction involves an imbalance in the production of vasodilator and vasoconstrictor mediators and a transition to a prothrombotic phenotype [79]. A hallmark of endothelial dysfunction is reduced NO bioavailability, which can be due to a reduced synthesis of NO or to an increased breakdown of NO secondary to ROS production [78].

Ceramide has been proposed to exert dual (protective or deleterious) effects on endothelial cells [65]. Thus, activation of eNOS following an acute exposition to TNF-α was shown to involve the activation of nSMase in HeLa and human endothelial cells [80,81]. Moreover, incubation with ceramide increases eNOS phosphorylation [80] and expression [65]. Despite these results, most evidence points to a negative effect of ceramide on endothelial function. In fact, short-term incubation with exogenous ceramide impairs endothelium-dependent vasorelaxation, a functional characteristic of endothelial dysfunction, in both systemic [32,33,72] and pulmonary arteries [31]. Furthermore, inhibition of ceramide production prevents endothelial dysfunction induced by palmitate [82] or lipopolysaccharide [31] suggesting a role for ceramide in the endothelial dysfunction induced by inflammation or obesity. In line with these evidences, Smith et al. [83] have shown that nSMase activity chronically increased with age leading to elevated ceramide and ultimately eNOS inactivation and lower NO synthetic capacity with age. Moreover a chronic production of nSMase-derived ceramide has been involved in the transition from NO to H_2_O_2_ as the primary endothelial-dependent mediator in coronary artery disease [70].

Ceramide may contribute to the development of endothelial dysfunction by either reducing the synthesis of NO or by increasing its breakdown secondary to ROS production. A reduction in eNOS activity by ceramide has been reported either under basal conditions or following agonist stimulation. Thus, ceramide facilitates the interaction of eNOS with its negative regulator caveolin-1 [84] and promotes the dissociation of the complex Akt/Hsp90/eNOS, thereby preventing the phosphorylation of eNOS at positive regulatory sites (Ser1177) and potentiating the phosphorylation of eNOS at negative regulatory sites (i.e., Thr495) [82,83,85,86,87]. Recently, the activation of protein phosphatase 2A (PP2A) has been proposed as a novel mechanism mediating the endothelial dysfunction following accumulation of ceramide in the context of type 2 diabetes and obesity [87,88].

In addition, ceramide has also been shown to induce endothelial dysfunction by increasing ROS production and reducing NO bioavailability [33,50,72,73,89]. Several studies have confirmed that ceramide is able to induce a rapid translocation of the NAPDH oxidase subunit p47phox and promote its interaction with the gp91phox subunits leading to NADPH oxidase activation [26,29,65,73,89]. However, other studies suggest that ceramide is able to exert direct effects on the mitochondrial ETC, leading to increased ROS production in several cell types, including endothelial cells [67,68,90]. The resulting O_2_^−^ can react with NO to form peroxynitrite (ONOO^−^), which in turn can lead to eNOS uncoupling leading to a self-amplifying deleterious network [50]. Finally, accumulating evidence suggests that the activation of the NLPR3 inflammasomes and the production of inflammatory cytokines induced by ceramide also play a major role in the development of vascular dysfunction and atherosclerotic lesions [31,91,92,93].

### 4.2. Ceramide and the Endothelial Barrier Function

Endothelial barrier dysfunction leading to increased permeability and plasma leak to surrounding tissues is a common hallmark of several inflammatory diseases including sepsis, systemic inflammatory response syndrome (SIRS), or acute lung injury [94]. Whereas S1P plays a key role maintaining endothelial barrier integrity, accumulation of ceramide increases endothelial permeability [95,96,97]. The ability of ceramide to increase endothelial permeability was first demonstrated by Goggel et al. [98] in the context of the platelet-activating factor (PAF)-induced lung oedema. Since then, ceramide has been proved to mediate the increase in vascular permeability induced by a wide range of inflammatory stimuli, including lipopolysaccharide (LPS) or cigarette smoke [31,95,96]. Ceramide disrupts the pulmonary endothelial barrier function by inducing direct apoptotic effects on endothelial cells [13], although mechanisms unrelated to its apoptotic effects have also been involved [98]. Thus, ceramide has been shown to disrupt tight junctions via activation of Rho kinase [96] but independent of p38MAPK [95]. In addition, ceramide has been proposed to trigger the failure of the endothelial barrier by the recruitment of TRPC6 channels and the subsequent increase in intracellular Ca^2+^ [95,99]. These effects could be amplified by the concomitant reduction of eNOS activity [84,99], as described in the previous section.

## 5. Ceramide as a Key Oxygen- and Mechano-Sensing Mediator

### 5.1. Ceramide and Oxygen Sensing

A number of cells are specialized in responding rapidly to changes in oxygen tension within the physiological range, playing a fundamental role in maintaining oxygen homeostasis [100,101,102,103]. Vascular smooth muscle cells from distal pulmonary arteries and from the ductus arteriosus (DA) belong to these specialized cell types that sense local O_2_ tension and respond to trigger vascular responses of crucial physiological relevance such as hypoxic pulmonary vasoconstriction (HPV) or normoxic DA constriction.

HPV is an adaptive physiological mechanism that allows ventilation/perfusion coupling [100,101,102,103]. While other factors may contribute, a great body of evidence strongly suggests that HPV is essentially an intrinsic feature of smooth muscle cells present in resistance pulmonary arteries in response to alveolar hypoxia [101]. The identification of the mechanisms involved in HPV has been a matter of intense debate during decades [101,102,103,104], with two main hypotheses being proposed. The redox hypothesis posed that the fall in O_2_ lead to a suppression of mitochondrial oxidative phosphorylation resulting in a more reduced cytosolic redox state and a decreased ROS production inducing the inhibition of Kv channels, membrane depolarization, and consequently the activation of voltage-gated Ca^2+^ channels and vasoconstriction [103,105]. Conversely the ROS hypothesis [106] proposed that hypoxia led to an increase in ROS (such as O_2_^−^ or H_2_O_2_), which mediate vasoconstriction by targeting multiple potential mechanisms such as voltage-dependent (through L-type channels) and voltage-independent (through TRPC6 channels) Ca^2+^ entry [107], Ca^2+^ release from ryanodine-sensitive stores, and Rho kinase-mediated Ca^2+^ sensitization among others [101,104]. The source of ROS during hypoxia has also been a matter of controversy since both mitochondria and the NADPH oxidases were involved and even a positive feedback between these two sources has been proposed [76].

**Figure 2:**
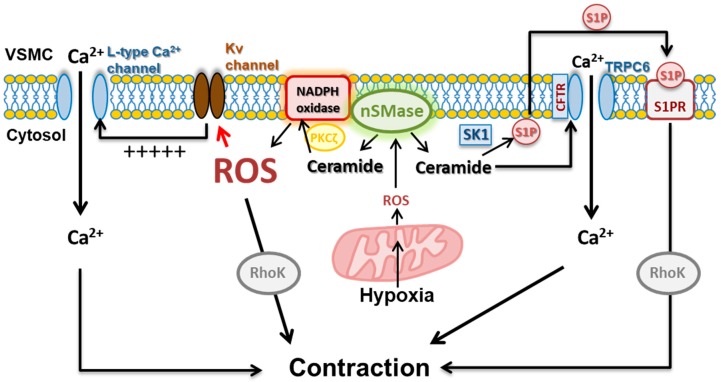
Schematic representation of the role of nSMase-derived ceramide in hypoxic pulmonary vasoconstriction (HPV). Hypoxia promotes mitochondria-derived ROS, which in turn stimulates the production of ceramide from nSMase. Ceramide amplifies ROS production through a PKCζ-dependent activation of NADPH oxidase. ROS inhibit Kv channels leading to membrane depolarization and activation of L-type Ca^2+^ channels, and activate RhoK-mediated Ca^2+^-sensitization. In addition, ceramide via CFTR stimulates Ca^2+^ entry through TRPC6 and, via a concomitant activation of SK1, leads to S1P generation which contributes to the contraction though the activation of RhoK. Black lines indicate a stimulatory effect, whereas the red line indicates an inhibitory effect. CFTR: cystic fibrosis transmembrane conductance regulator; Kv: voltage-gated K^+^ channels; nSMase: neutral sphingomyelinase; PKCζ: protein kinase Cζ; RhoK: Rho-kinase; ROS: reactive oxygen species; S1P: sphingosine-1-phosophate; S1PR: sphingosine-1-phosophate receptor; SK1: sphingosine kinase 1; VSMC: vascular smooth muscle cell. Black arrows indicate activation and red arrow indicates inhibition.

A decade ago, our group identified ceramide as a key player in HPV [27]. We provided evidence that the HPV responses ex vivo and in vivo require nSMase- but not aSMase-derived ceramide production. Hypoxia was shown to induce a rapid increase in ceramide within the pulmonary artery smooth muscle cells and this led to the inhibition of Kv currents [27], in line with the redox theory. However, this pathway was associated with an increase, rather than a decrease of ROS, according to the ROS hypothesis [26]. Hypoxia-induced ceramide production was prevented by the mitochondrial ETC inhibitor rotenone [26], in agreement with the proposal of the O_2_ sensor being located in mitochondria [100,106,108]. Interestingly, hypoxia-induced ROS production, Kv current inhibition and vasoconstriction were prevented by inhibitors of both mitochondrial ETC and NADPH oxidases [26]. Additionally, ceramide through its canonical target PKCζ [109] rapidly activated NADPH oxidase (via p47phox phosphorylation) to increase ROS production [26]. Altogether, these data led to a proposal of an integrated signaling pathway for HPV which included the mitochondrial ETC as the sensor and nSMase-PKCζ-NADPH oxidase as a necessary redox amplification pathway required for ROS production and vasoconstriction (Figure 2). Interestingly, activation of this pathway was shown to contribute to TXA_2_- (but not endothelin-1) induced pulmonary vasoconstriction [28]. In fact TXA_2_-induced vasoconstriction is associated with activation of PKCζ [39,110,111], ROS generation via NADPH oxidase [47], and inhibition of Kv currents [39,110,111]. The proposal of nSMase-PKCζ-NADPH oxidase as an amplification mechanism in HPV is in agreement with the ROS-induced ROS production mechanism reported for HPV [76] and with the dual role of ceramide as a product (being generated by redox-sensitive nSMAse) or as a trigger (by forming redox signaling platforms) of ROS production. Further evidence of the role of nSMase-derived ceramide in HPV was provided more recently by Tabeling et al. [40]. These authors showed that activation of nSMase by acute hypoxia leads to the translocation of TRPC6 to caveolae, contributing to calcium mobilization and pulmonary vasoconstriction (Figure 2). TRPC6 recruitment to caveolae required the role of cystic fibrosis transmembrane conductance regulator (CFTR). As previously shown [27,107,112], an increase in calcium sensitization by Rho kinase activation was also shown to contribute to HPV, although the conversion from ceramide to S1P could account for this effect [40]. Remarkably, ceramide-induced pulmonary vasoconstriction mimics HPV mechanistically as it is triggered through inhibition of Kv channels, activation of TRPC, or activation of Rho kinase [27,28,40].

HPV represents a unique response of pulmonary arteries since in systemic arteries hypoxia induces vasorelaxation. Waypa et al. [108] showed that under hypoxia smooth muscle cells from both pulmonary or systemic arteries exhibited increases in mitochondrial-derived ROS, but functional responses were opposite (smooth muscle cells from pulmonary arteries exhibited an increase in intracellular Ca^2+^ while those from systemic arteries showed a decrease), and suggested the role of cell-specific expression of downstream signaling pathway. It is worth highlighting that hypoxia fails to generate ceramide in mesenteric arteries [27], suggesting the specificity of the nSMase pathway, that could serve as an amplification ROS signal in pulmonary but not in systemic arteries. The selective increased ceramide production in oxygen sensing vessels could be related to higher nSMase expression (i.e., nSMase2, [27,29]). Also, a closer localization of mitochondria to the plasmalemmal membrane in these cells [113] could facilitate the functional coupling between mitochondrial ROS and nSMase activation.

During fetal life, the DA connects the pulmonary artery with the aorta allowing venous blood to bypass the nonventilated lungs. Due to the fetal hypoxic environment the DA is tonically relaxed. At birth the increase in O_2_ tension is a key factor stimulating DA constriction, which precedes the anatomic and permanent closure of the vessel to establish the postnatal pattern of circulation [103,114]. A number of mechanisms have been reported to contribute to O_2_-induced DA constriction. Michelakis et al. [115] showed that O_2_-induced constriction was mediated through the inhibition of Kv channels, resulting in membrane depolarization, and Ca^2+^ entry through voltage-operated Ca^2+^ channels. Thereafter, these authors proposed a model in which the increase in O_2_ was sensed by the mitochondrial ETC (the sensor), leading to an increased production of ROS (i.e., H_2_O_2_, the mediator) resulting in Kv channel inhibition (the effector) [116]. Further studies confirmed the role of Kv channel inhibition [117,118] and identified additional downstream effectors of the O_2_-sensing system such as Ca^2+^ sensitization of contractile proteins induced by Rho kinase activation or Ca^2+^ entry though TRPC channels [117,119,120]. Of note, the mechanisms involved in DA constriction resemble those for HPV despite being diametrically opposed stimuli suggesting the existence of a common signaling. Using a chicken embryo model, Moreno et al. [29] demonstrated that ceramide content was rapidly increased by hypoxia in pulmonary arteries and by normoxia in the DA. Likewise inhibition of nSMase using siRNA and pharmacological approaches markedly reduced HPV and normoxic DA contraction, strongly suggesting the role of ceramide as a common mediator in both responses. Remarkably, the involvement of nSMase-ceramide in acute oxygen sensing was also confirmed in human pulmonary arteries and DA [29].

Altogether, these studies are consistent with a prominent role of nSMase-derived ceramide in oxygen sensing in vascular tissues.

### 5.2. Ceramide and Mechano-Sensing

Vasodilation to shear stress (flow-induced dilation) is an important physiological mechanism linking vascular tone with the dynamics of fluid flow, which involves the endothelial production of vasoactive mediators (especially NO). Czarny M et al. [121] reported an acute mechanoactivation of nSMase in response to increased pulmonary blood flow, which was associated with an increase in ceramide content at the luminal endothelial cell surface primarily in caveolae. In a subsequent study, these authors showed the activation of the Akt/eNOS pathway by acute exposure to ceramide [122]. However, the role of nSMase-derived ceramide in flow-induced NO production or vasodilation has not been confirmed and even a potential deleterious effect of ceramide on flow-induced dilation has been proposed. Thus, Freed et al. [70] showed that chronic exposure to ceramide switched the vasoactive mediator of flow-induced dilation from NO to deleleterious reactive oxygen species such as H_2_O_2_, resembling what occurs in arterioles from coronary artery disease (CAD) patients. More recently, the formation of endothelium-derived extracellular vesicles by ceramide has recently been proposed as a mechanism leading to this transition [123]. Remarkably, inhibition of nSMase-derived ceramide formation in arterioles from CAD patients was shown to reverse the mediator of flow-induced dilation from the pro-atherogenic H_2_O_2_ to NO, which is athero-protective [70]. Since flow-induced dilatation is a key determinant of myocardial blood flow distribution and its deterioration is a predictor of cardiovascular events, these studies suggest nSMase as a potential therapeutic target for pharmacological intervention and ceramide as a possible biomarker in the context of CAD.

## 6. Ceramide and Disease

Ceramides are essential components of every cell membrane and, therefore, any imbalance in their metabolism or the tissue accumulation of ceramide species may result in the activation of a number of different signaling pathways, many of which are detrimental to normal cellular function. A good example of the diversity of the potential deleterious effects of alterations in ceramide homeostasis is Farber disease (OMIM #228000). Farber disease is caused by mutations in *ASAH1* gene, which lead to decreased acid ceramidase activity and, in turn, to ceramide accumulation in almost every tissue of the body [124,125]. Farber disease has a heterogeneous presentation ranging from a severe phenotype with respiratory and neurological involvement and a very short life expectancy to a moderate phenotype, which generally includes joint swelling, contractures, and pain [124,125]. Besides these main symptoms, gastrointestinal, hepatological, cardiovascular, ophthalmological, dermatological, hematological, neuromuscular, and bone alterations are described in patients with Farber disease [124,125,126].

The evidence on the potential or actual involvement of ceramide metabolism in the etiopathogenesis of a growing number of conditions has been summarized and discussed in several very recent reviews and editorial comments. These conditions include, among others, cancer [10,127,128], neurological, neurodegenerative, and psychiatric disorders [7,15,57,129,130], infection/inflammation [131,132,133,134], metabolic conditions [135,136,137,138], cardiovascular disease [139,140,141,142], eye disease [143], skin disease [144,145], and lung disorders [4,14]. Although an exhaustive review is beyond the scope of this article, we will provide a brief description of the most relevant evidence from human studies on the role of ceramide in cardiovascular and pulmonary conditions.

### 6.1. Ceramide and Cardiovascular Disease. The Role of Metabolic Syndrome

Over the past few years an increasing number of studies have emerged revealing the association of circulating ceramide levels with adverse cardiovascular events such as myocardial infarction and stroke [140,141]. These studies consistently show that a subset of ceramides with long and very long chains (e.g., C16:0, C18:0, C20:0, C22:0, C24:1) almost invariably associate with deleterious outcomes and this association was independent of plasma lipid markers and other traditional cardiovascular risk factors [12,146,147,148,149,150,151] (Table 2). In contrast, C24:0 show no or negative relationships with adverse cardiovascular events. The ratio of the “harmful” ceramides against the “benign” C24:0 species has been proposed to be incorporated in the arsenal of biomarkers that predict cardiovascular disease [140,141].

The identification of the molecular mechanisms by which some particular ceramides drive cardiovascular dysfunction has received considerable attention but remains largely unknown. An important part of the link between ceramide and cardiovascular disease may operate through the metabolic syndrome [138]. The metabolic syndrome is a cluster of interconnected physiological, biochemical, clinical, and metabolic factors linked to an increased risk of cardiovascular diseases and type 2 diabetes mellitus [154]. Elevated blood pressure, atherogenic dyslipidemia (increased triglycerides and reduced high-density lipoprotein cholesterol), endothelial dysfunction, hypercoagulable state, insulin resistance, central obesity, and chronic stress are the several factors which constitute the syndrome [154]. Therefore, obesity, insulin resistance, type 2 diabetes mellitus, and cardiovascular disease form a pathologic continuum in which ceramide may be one of the most relevant connecting mediators through its capacity of disrupting insulin sensitivity, pancreatic β cell function, vascular reactivity, and mitochondrial metabolism [138,155].

Despite the influence of dietary intake on the circulatory levels of lipids, plasma levels of lipid species are found to be heritable, and ceramides showed the greatest estimated heritability [156]. In addition, mutations in ceramide-modifying genes have been shown to associate with glycosylated hemoglobin (HbA1c), the most reliable marker of chronic hyperglycemia, [157] and increased risk of arterial and venous thrombosis in humans [156], and there is confirmatory evidence from relatively large human cohorts on the relationships between serum ceramides and insulin resistance [158,159] (Table 3). Abundant experimental evidence from rodent models shows that inhibition or ablation of the enzymes involved in ceramide biosynthesis, as well as stimulation of ceramide degradation, are insulin sensitizing, antiatherogenic, and cardioprotective [138]. There is also experimental evidence on the role of different ceramide species in several atherosclerotic processes such as aggregation of lipoproteins, accumulation of lipoproteins and cholesterol within macrophages and vessel wall, impairment of mitochondrial function leading to excessive production of ROS, regulation of NO synthesis, activation of platelets, and expression of various cytokines [70,134,142,155]. Moreover, ceramides also modulate signaling and metabolic pathways involved not only on insulin resistance, but also on hepatic steatosis, and hypertension and, therefore, play a key role in the metabolic dysfunction that precedes cardiovascular events [138,139,140,160,161]. Finally, some ceramides may trigger cardiomyocyte-specific actions leading to the so termed lipotoxic cardiomyopathy [155,160,162].

### 6.2. Ceramide and Lung Diseases

A growing body of evidence suggests an important role of sphingolipids in lung diseases, including pulmonary hypertension (PH), acute respiratory distress syndrome (ARDS), chronic pulmonary obstructive disease (COPD), asthma or cystic fibrosis, as reviewed elsewhere [4,14,163,164]. In this review, we will focus on those diseases with a direct effect on the pulmonary vascular circulation.

ARDS is characterized by the development of pulmonary edema which causes alveolar collapse and HPV failure leading to severe arterial hypoxemia. Furthermore, some patients develop mild to moderate PH which is independently associated with poor outcomes in patients with ARDS [165,166] (Table 4). Patients in critical care show increased levels of ceramide-generating enzymes, including aSMase [167], and the role of ceramide in the development of pulmonary edema is well stablished in both patients and experimental models [98,168,169,170]. Furthermore, our group has recently described a potential association between ceramide and HPV failure in the context of ARDS [31]. Thus, a growing number of evidence appears to confirm that increased production of ceramides act as broad markers of lung injury.

However, clinical studies have uncovered a potential conflicting role of ceramide in acute versus chronic lung diseases. A good example can be found in the context of bronchopulmonary dysplasia (BPD). BPD is the most prevalent chronic lung disease during infancy, its incidence increases with the degree of prematurity and the pathology is characterized by a disruption of vascular development and alveolarization, with long term consequences [182]. PH develops in up to 40% of these patients and it is associated with a worst prognosis. Although our group has demonstrated the involvement of nSMase-derived ceramide in oxygen sensing during the neonatal period [29], only a limited number of studies have analyzed the potential link between sphingolipids and the risk of developing BPD. These studies consistently reported an increased in the levels of ceramide in tracheal aspirates in pediatric patients undergoing mechanical ventilation [172,173]. However, ceramide production tends to increase with postnatal development and this increase shows no or even a negative correlation with long term complications [171,172].

PH refers to a heterogeneous group of diseases, with a significant morbidity and mortality, which are characterized by a sustained elevation in pulmonary arterial pressure [183]. Vasoconstriction, remodeling of the pulmonary vessel wall, inflammation and thrombosis contribute to the increased pulmonary vascular resistance in PA in these patients. A link between the dysregulation of the ceramide/S1P rheostat and the development of PH has also been proposed. Thus, ceramide and/or S1P are involved in all the features associated with the development of PH, including vasoconstriction [27,29,31,40], inflammation [31] and proliferation of pulmonary artery smooth muscle cells [184]. In line with this, inhibition of SK1 prevents the development of hypoxia-induced PH in rodents [174]. However, despite these promising results, clinical data are still scarce on this topic. To the best of our knowledge, only a limited number of studies have analyzed the levels of ceramide and S1P in a limited number of patients with pulmonary arterial hypertension (PAH) (Table 4). Although these studies have not detected significant differences in the pulmonary levels of ceramide, an increase in S1P has been confirmed in both peripheral blood samples [185] and lung tissues from patients with PAH [174,184,186]. Notably, a recent report [175] suggests that ceramide levels are increased in the right ventricle of patients with PAH. These novel findings suggest that, in addition to pulmonary vascular abnormalities, accumulation of ceramide in the right ventricle may also contribute to the leading cause of death in PAH, the failure of the right ventricle.

The development of PH is also a key determinant of poor prognosis in patients with COPD and cystic fibrosis [187,188]. nSMase is increased in the lungs from patients with emphysema [189] and increased ceramide production is found in lungs from patients with COPD or cystic fibrosis [178,180]. However, the levels of ceramide in the lungs were not found to be associated with the degree of severity of the disease [179,180]. In contrast, a negative correlation between plasmatic levels of ceramide and the development of emphysema has recently been found [181].

Although many questions remain to be answered, including the relative contribution of the ceramide/S1P rheostat in specific tissues (i.e., the lungs vs. the right ventricle or the systemic circulation), an increasing number of evidences suggest that the modulation of sphingolipids are a promising therapeutic target in pulmonary vascular diseases.

## 7. Conclusions

The present review provides consistent evidence of the role of ceramide in regulating vascular function. Ceramide exerts variable effects on vascular tone that reflect its involvement in a number of signaling pathways in different vascular cells. In particular, the vascular production of ceramide is closely related to the redox state, serving as amplification mechanisms of ROS signaling in physiological (oxygen sensing) or pathological (endothelial dysfunction) responses.

In recent years, the potential significance of ceramide in a wide variety of pathological entities has been investigated. There is still a considerable amount to be learned regarding ceramide signaling in vascular tissues, especially regarding the different ceramide subspecies, their source, their intracellular compartmentalization, and their participation in specific vascular responses.

## Figures and Tables

**Table 1:** Summary of the vasomotor effects induced by ceramide. SMases: sphingomyelinases; KCa: calcium-activated K^+^ channels; ROS: reactive oxygen species; PKCζ: protein kinase Cζ.

Stimulus	Vascular Effect	Preparation	Mechanism	Ref.
C2-ceramide	Transient vasodilation	Rat mesenteric arteries	Activation of Guanylyl cyclase, KCa channels	[20]
C2-ceramide	Vasodilation	Rat aorta	Endothelium-derived NO	[21]
C2-ceramide	Vasodilation	Rat aorta	Endothelium-independent	[22]
C2-ceramide, SMase	Vasodilation	Rat aorta	Inhibition of RhoA/Rho kinase and Ca entry	[23]
C2-ceramide	Vasoconstriction	Small bovine coronary arteries	Inhibition of Kca	[24]
SMase, C8 and C16-ceramide	Vasoconstriction	canine cerebral arteries	PKC activation and Ca entry	[25]
SMase C6-ceramide	Vasoconstriction	Rat pulmonary arteries	PKCζ activation, Rho kinase activation and decreased Ca entry	[26,27]
SMase C6-ceramide	Vasoconstriction	Rat and human pulmonary arteries	PKCζ activation and Kv channel inhibition	[28]
SMase C6-ceramide	Vasoconstriction	Chicken and human pulmonary arteries and ductus arteriosus	PKCζ activation and Kv channel inhibition	[29]
C2-ceramide	Enhanced vasoconstriction	Rat aorta	Endoplasmic reticulum stress	[30]
SMase	Reduced vasodilation Increased contraction to 5-HT	Rat pulmonary arteries	IL-6	[31]
C2-ceramide	Attenuated vasodilation	Bovine coronary arteries	Increased ROS	[32]
C2-ceramide	Attenuated vasodilation	Mice carotid arteries	Increased ROS	[33]
C6, C24:1 and C24:0 ceramide	None	Canine cerebral arteries		[25]

**Table 2:** Plasma levels of ceramide and cardiovascular disease.

Study	Population	Main Findings
Tarasov et al. 2014 [146] LURIC	Males (258) with coronary artery disease who died within 3 years of follow-up and 187 matched control patients with coronary artery disease who did not die during the follow-up.	Higher plasmatic levels of C16:0, C18:0, C20:0, and C24:1, and lower levels of C24:0 in patients who died. Ratios of C16:0 /C24:0 and C22:0/C24:0 were significantly related to increased risk of death in all subjects and subgroups. C24:0/C24:1 was indicative of a reduced risk of death, regardless of diabetes status. Simvastatin lowered plasma ceramides.
Alshehry et al. 2016 [152] ADVANCE	Cohort of 3799 individuals with type 2 diabetes that included 698 patients with cardiovascular events and 355 with cardiovascular death.	Significant association between C24:1 and death.
Havulinna et al. 2016 [147] FINRISK 2002	Individuals (8101) from the FINRISK 2002 general population cohort (men and women aged 25 to 74 years). During a follow-up of 13 years, 813 subjects experienced an incident major adverse cardiovascular event, of which 116 were fatal.	Levels of C16:0, C18:0, and C24:1 and the ratios C18:0/C24:0, and C24:1/C24:0 were significantly higher in subjects with an incident major adverse cardiovascular event compared with asymptomatic subjects. C18:0 holds the potential for improving the risk classification over the Framingham risk score at a population level. C16:0 and C24:1 associated with recurrent major adverse cardiovascular event.
Wang et al. 2017 [148] PREDIMED	Participants (980) from the PREDIMED trial (Prevención con Dieta Mediterránea), including 230 incident cases of cardiovascular disease and 787 randomly selected participants at baseline.	Plasma concentrations of C16:0, C22:0, C24:0, and C24:1 were positively associated with incident cardiovascular disease risk.
Mantovani et al. 2018 [149]	Patients (581) with established or suspected coronary artery disease undergoing stress myocardial perfusion scintigraphy.	Higher plasmatic levels of C18:0, C20:0, C22:0, and C24:1 were associated with lower post stress anteroapical wall perfusion. Associations persisted after adjustment for conventional cardiovascular risk factors.
Anroedh et al. 2018 [150] ATHEROREMO	Patients (581) undergoing diagnostic coronary angiography or percutaneous coronary intervention for stable angina pectoris (SAP) or acute coronary syndrome (ACS).	C16:0 concentration was associated with major adverse cardiac events after adjustment for cardiac risk factors, clinical presentation, statin use, and HDL cholesterol level. After multivariable adjustment, concentrations of C16:0, C20:0, C24:1 and their ratios to C24:0 were associated with the composite endpoint death or nonfatal acute coronary syndrome.
Meeusen et al. 2018 [12]	Patients (504) between 18 and 75 years of age, who were undergoing clinically indicated coronary angiography.	Concentrations of C16:0, C18:0, and C24:1 and their ratios to C24:0 were significantly predictive for a combined outcome of myocardial infarction, coronary artery bypass graft, percutaneous intervention, stroke, and death at 4 years of follow-up. Concentrations of C16:0, C24:1 and the ratios C16:0/ C24:0, C18:0/ C24:0, and C24:1/C24:0 were significantly predictive for all-cause death at 18 years of follow-up and remained significant after adjusting for cardiovascular risk factors.
de Carvalho et al. 2018 [153]	Two cohorts of patients (337 and 119) with acute myocardial infarction, undergoing coronary angiography.	Identification of a 12-ceramide plasma prognostic signature that predicted long-term major adverse cardiac and cerebrovascular events in patients with acute myocardial infarction, of which C22:1, C24:1 and dihydro C16:0 were the strongest predictors.

**Table 3:** Plasma levels of ceramide and insulin resistance.

Study	Population	Main Findings
Wigger et al. 2017 [158]	Individuals (288) from the D.E.S.I.R. cohort study (Data from Epidemiological Study on the Insulin Resistance syndrome)	The susceptibility to develop type 2 diabetes mellitus was associated with increased plasma levels of C18:0, C20:0, C22:0, and dihydro C22:0
Lemaitre et al. 2018 [159]	Cohort of 2086 Native Americans without diabetes (average age of 38 years), 24% of whom had a BMI of 35 kg/m^2^ or greater.	Participants with higher (>90th percentile) plasmatic levels of C16:0, C18:0, C20:0, or C22:0 displayed hyperinsulinemia and insulin resistance

**Table 4:** Ceramide and lung diseases.

Study	Population	Main Findings
*High altitude pulmonary edema (HAPE)*		
Guo et al. 2015 [168]	22 HAPE subjects and 22 healthy controls	Plasma levels of C8-ceramide (and sphingosine) are increased in HAPE subjects compared to subjects not developing any symptoms after exposure to high altitude.
*Bronchopulmonary dysplasia (BPD)*		
Laube et al. 2017 [171] van Mastrigt et al. 2018 [172]	43 preterm infants (24–28 weeks): 25 received inhaled NO (iNO) and 18 placebo. 122 preterm babies (<32 weeks): 41 developed BPD.	aSMase activity in tracheal aspirates (TA) increases during the first two weeks of life and this increase is more pronounced in infants receiving iNOS. Ceramide levels are increased in TA following mechanical ventilation. Ceramide profiles changed over time with mechanical ventilation. Ceramide levels were lower in infants who developed BPD as compared to those who did not.
*Congenital diaphragmatic hernia (CDH) and development of chronic lung disease*		
Snoek et al. 2016 [173]	72 neonates (>34 weeks) with CHD.	Higher levels of ceramides (C18:1 and C24:0) in TA were found in patients in conventional mechanical ventilation compared to high-frequency oscillation. Levels of ceramides were not associated with a higher risk of mortality or chronic lung disease.
*Pulmonary arterial hypertension (PAH)*		
Chen et al. 2014 [174] Brittain et al. 2016 [175]	5 PAH patients and 5 control subjects. 19 PAH patients and 22 control subjects.	In lungs from PAH patients, levels of ceramide remains unchanged but levels of S-1P and its receptor S1PR2 are increased. Ceramides C16:0 and C24:0 were increased in right ventricle from PAH patients.
*Cystic fibrosis (CF)*		
Guilbault et al. 2009 [176] Garic et al. 2017 [177] Brodlie et al. 2010 [178]	58 patients with CF and 72 healthy control subjects. 15 CF patients and 15 health volunteers. 24 patients undergoing lung transplantation with CF (8), emphysema (8) or PH (8).	Plasma levels of ceramide were decreased in patients with CF. Plasma levels of C22:0, C24:0 and C26:0 were decreased in patients with CF whereas C16:0 was increased. Ceramide staining was increased in lungs from patients with CF or emphysema as compared to those from PAH patients or unused donors. C16:0, C18:0, and C20:0 (but not C22:0) were significantly increased in lungs with CF vs. PAH.
*Chronic obstructive pulmonary disease (COPD)*		
Lea et al. 2016 [179] Scarpa et al. 2013 [180] Bowler et al. 2015 [181]	10 patients with COPD and 7 controls (5 smokers). 35 patients with COPD and 11 nonsmoking controls. 250 patients with COPD, 122 of them with emphysema.	Levels of C16:0, C18:0 and C20:0 might be increased in alveolar macrophages from COPD patients. Lung ceramide was increased in COPD patients but was not related to the severity of the disease. Plasma ceramide levels were inversely correlated with emphysema severity whereas trihexosylceramide positively correlated with COPD exacerbations.
